# Motility of select ovarian cancer cell lines: Effect of extracellular matrix proteins and the involvement of PAK2

**DOI:** 10.3892/ijo.2014.2553

**Published:** 2014-07-22

**Authors:** ELIZABETH FLATE, JOHN R.D. STALVEY

**Affiliations:** 1 Kent State University, Kent, OH 44242, USA; 2 University of North Carolina, Chapel Hill, NC 27514, USA; 3 University of Alaska, Anchorage, AK 99508, USA

**Keywords:** cell migration, metastasis, ovarian cancer, extracellular matrix, p21-activated kinase 2

## Abstract

The interaction between tumor cells and extracellular matrix (ECM) proteins influences cell migration and the invasive behavior of cancer cells. In this study, we provide experimental evidence that collagen I and fibronectin affect ovarian cancer cell migration. *In vitro* wound healing assays and transwell migration assays were used to measure both total wound healing and directionality of individually migrating OV2008 and C13 ovarian cancer cells on glass, collagen I and fibronectin. Involvement of p21-activated kinase 2 (Pak2) in the motility of these cell lines was investigated using a chemical inhibitor as well as siRNA transfection. Culturing ovarian cancer cells on collagen type I (COLL) increased the migratory ability of OV2008 and C13 cells by increasing the directional migration of cells. In contrast, fibronectin (FN) decreased the migratory ability of OV2008 cells by reducing their ability to migrate directionally. When both cell lines are cultured on COLL and treated with increasing concentrations of a PAK inhibitor (IPA-3), there is a dose-dependent response such that there is a decrease in migration with an increase in inhibitor concentration. Further experiments utilizing PAK2 knockdown via siRNA transfection demonstrated significantly reduced migration of OV2008 cells on COLL as compared to those receiving control siRNA. In conclusion, our results indicate that FN and COLL affect the motility of the selected ovarian cancer cells lines and the effect of COLL is likely mediated, at least in part, by PAK2. A better understanding of the molecular events that contribute to tumor invasion and metastasis is crucial for developing novel treatment strategies to improve the long-term survival of women with ovarian cancer. As PAK2 is involved in motility, it should be further explored as a pro-metastatic gene in ovarian cancer.

## Introduction

Cell migration pathways play significant roles in a variety of physiological processes that can be ‘hijacked’ by cancer cells and thus, increase the metastatic potential of the disease and increase the incidence of cancer-related deaths (1). Regardless of when it occurs, the dissemination of tumor cells from the primary tumor is the principle reason for the mortality and morbidity of cancer patients. This appears to be particularly important for ovarian surface epithelial (OSE) cancer, which is often diagnosed only after cells have been released into the peritoneal cavity. Therefore, investigation into the molecular mechanisms of tumor cell motility has been intensified. Therapies that specifically target the motility of tumor cells could significantly improve cancer treatment by removing the threat of systemic disease and decreasing the sole dependence on cytotoxic therapeutics with detrimental side-effects (2).

The dynamic interaction between a cell and extracellular matrix (ECM) proteins influences cell migration and the invasive behavior of cancer cells. These proteins influence both the motility and migratory capacity of normal cells, and the metastasis of tumor cells. Although the extracellular matrix was initially perceived as a barrier to tumor cell dissemination, it is becoming evident that the architecture and the matrix composition of the microenvironment can promote tumor cell dissemination. Specifically ECM proteins have been shown to influence movement in ovarian cancer cells through interaction with integrins (3).

As groups, integrins mediate adhesion and mechano-transduction to extracellular ligands via α2β1 integrin predominantly binding to fibrillar collagen; αvβ3, αvβ1 and α5β1 interacting with fibronectin; and α3β1 and α6β1 engaging with laminin (4). After associating with ligands, the cytoplasmic tails of integrins interact with cytoskeletal adaptor proteins (5,6). Adaptor and mechanosensing modulator proteins engage with the actin cytoskeleton and trigger signaling to protein kinases, including focal adhesion kinase (FAK) and Src (5,7,4). Downstream integrin effectors further include the small GTPases Rac and Rho, which reinforce cell protrusion and rear contraction (8). The Rho-family GTPases have been directly linked to motility and protrusion formation through their ability to activate the signaling targets that regulate actin cytoskeleton modification (9).

Cell migration can be subdivided into random motility and directed migration, the latter indicating whether a cell is able to maintain a single direction of migration for prolonged periods of time (10). Directional motility requires polarization, and maintaining polarity determines persistence with which a cell moves directionally. Morphological polarization is a consequence of an internal asymmetry in distribution of signaling molecules and cellular structures, primarily cytoskeletal (11). External stimuli are not necessarily required to trigger and maintain polarity, although they can contribute and shift equilibria in favor of polarized states. Chemotactic and haptotactic gradients serve as external guides by keeping up cell polarity and reducing the probability of changing direction. Polarized motility is governed by organization of a leading edge in the direction of cell movement. The leading edge is stabilized by the formation of new focal adhesions or cell-ECM contact sites (12). While directional cell migration facilitates the coordinated movement of cells during the development and tissue repair, the pathways involved with regulating the interplay between the extracellular environment, the actin cytoskeleton and the plasma membrane that result in directional movement remain inadequately understood.

It is believed that intracellular signaling, often mediated at the leading edge by the Rho family of small GTPases, operates at each step of the cell motility cycle to promote directional migration by regulating leading edge formation. Among the downstream targets of the Rho family of small GTPases, PAK (p21-activaed kinases) serine/threonine protein kinases have been implicated as effectors of cell motility. These kinases are subdivided into two groups, PAK1-3 (group I) and PAK4-6 (group II). The group I isoforms contain an autoinhibitory domain (PID) which is absent in group II PAK proteins (13). The group I PAKS share a p21-binding domain (PBD), a serine/threonine kinase domain, an acidic region and multiple proline-rich regions that serve as binding sites for SH3 domain-containing proteins. Pak kinases were first identified in screens for Rac and Cdc42 effectors (14).

Several targets of Paks are directly implicated in regulating cytoskeletal dynamics, including LIM domain kinase 1 (15), which phosphorylates and inactivates cofilin, an F-actin-severing and -depolymerizing protein, or myosin light chain (16) and MLC kinase (17), which control myosin contractility. Paks are also involved in the reorganization of the focal adhesions (18,19). So it appears logical that PAK(s) could play important role(s) in modulating the ability of cancer cells to move and metastasize. A number of human breast cancer lines exhibit constitutively elevated PAK1 and PAK2 activity, in some cases associated with the presence of an activated Rac GTPase (20) and PAK activity has been linked to tumor invasiveness and motility of a variety of human cancer cell lines (21).

A better understanding of motility and its role in cancer metastasis is crucial to reducing the number of annual deaths due to metastatic disease. Therefore, the experiments reported herein were designed to test the hypothesis that ECM proteins (specifically collagen I and fibronectin abundant in the peritoneal mesenteries) are important in ovarian cancer metastasis, facilitating cellular motility. We identified Pak2 as a possibly important mediator of ovarian cancer cell migration on ECM.

## Materials and methods

### Materials

Fetal bovine serum (FBS) was obtained from Innovative Research (Novi, MI). RPMI-1640 media with L-glutamine and without sodium bicarbonate was purchased from HyClone Laboratories, Inc. (Logan, UT). CellTiter 96^®^ AQueousOne Solution Cell Proliferation Assay (MTS) was purchased from Promega (Madison, WI). Antibiotic antimycotic solution (100X) stabilized, with 10,000 units penicillin, 10 mg streptomycin and 25 μg amphotericin B per ml (PSA) was obtained from Mediatech, Inc. (Manassas, VA). Human plasma fibronectin and bovine collagen type I was ordered from BD Biosciences (Franklin Lakes, NJ). Sodium bicarbonate, HEPES and trypsin solution from porcine pancreas were purchased from Sigma-Aldrich (St. Louis, MO). T-25 cm^2^ cell culture flasks were produced by Santa Cruz Biotechnology, Inc. (Santa Cruz, CA).

### Cell lines and culture conditions

The cisplatin-sensitive cell line, OV2008, was derived from ovarian serous cystadeno-carcinoma from a patient without prior chemotherapy (22); and its resistant variant, C13, originated from *in vitro* cisplatin challenges of OV2008 cells (23). These cells were acquired as a gift from Dr Barbara Vanderhyden (University of Ottawa, Canada). The OV2008 and C13 cell lines were maintained in RPMI-1640 (1X) medium, supplemented with 10% fetal bovine serum (FBS) and 1% PSA, sodium bicarbonate (24 mM) and HEPES (10 mM). All cell cultures were maintained at 37°C in a humidified atmosphere with 5% CO_2_.

All wound healing assays were performed in modified 35-mm cell culture dishes. These dishes were created by punching a hole in the bottom of the dish followed by adherence of a 22-mm^2^ glass cover slip (Corning) to the bottom of the dish. These dishes were baked at 60°C for 2 days before being soaked overnight in a CytoClean solution. The dishes were then rinsed, dried and sterilized via exposure to UV light for 2.5 h.

### Culture of ovarian cancer cell lines on collagen I and fibronectin

The substrata that were used in the current investigation were selected to represent some of the different types of ECM that OSE cells may contact, *in vivo*, when disseminated into the peritoneal cavity. Glass cover slips were coated with extracellular matrix proteins by suspending the ECM proteins in a solvent (fibronectin in PBS, collagen I in 0.1 N HCl). Collagen was used as a thin coating at 10 μg/cm^2^ and fibronectin was applied at a 5-μg/cm^2^ as per the manufacturer’s recommendations. The ECM solution was then added to the cover slips and allowed to incubate for 1 h at room temperature. The cover slips were then quickly rinsed with PBS before plating the cells onto the coated cover slips.

### Wound healing assay

**Figure 1:**
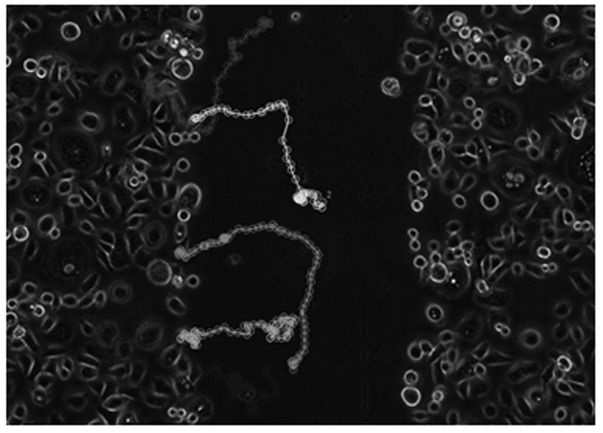
Tracking of individual cell movement during *in vitro* time-lapse recorded wound healing assay.

Cell migration was measured using an *in vitro* wound healing assay. OV2008 and C13 cells were allowed to form a confluent monolayer in modified 35-mm tissue culture dishes until confluent. The wound was created by scraping monolayer cells with a sterile pipette tip to scratch a ‘wound’ into the confluent monolayer. The media was changed to remove debris and cells. The dish was placed into a stage top incubation LiveCell device (Pathology Devices, Exton, PA). The LiveCell device maintained the temperature at 37°C, the CO_2_ at 5%, and the relative humidity at 75% within the stage top chamber. Slidebook software was used to take a picture at time point zero and every 10 min for a total of 10 h using an Olympus IX70 inverted microscope (Center Valley, PA). TScratch software (developed by Tobias Gebäck and Martin Schulz, ETH Zürich) was used to analyze the images, measuring the differences in migration. Values are presented as percentage (%) of open area (‘wound’) remaining at 10 h compared to 0 h. The time lapse stacks of images were also analyzed using ImageJ and the two following plug-ins: i) Manual Tracking (developed by Fabrice Cordeli, Institute Curie, Orsay, France) and ii) Chemotaxis and Migration Tool (Ibidi, Martinsried, Germany). Individual cells were randomly selected and tracked throughout the 10-h time period, as demonstrated in Fig. 1.

### Migration assay

OV2008 and C13 cells were grown in 35-mm tissue culture dishes until confluent. Cells were then trypsinized and migration assays were performed using ThinCerts migration inserts with 8 μm pore size (Bioexpress, Kaysville, UT). Briefly, 2×10^5^ cells suspended in 200 μl of serum-free RPMI were added to the upper compartment of the insert, which rests in the well of a 24-well plate. RPMI (650 μl) containing 10% FBS was added to the bottom compartment with serum providing the chemoattractant signal. The cells were cultured at 37°C and 5% CO_2_ and allowed to migrate for 24 h. The inserts were removed and the remaining non-migrating cells on the upper surface of the membrane were removed with a cotton swab. The cells that migrated to the lower surface of the membrane were fixed with 4% formaldehyde for 5 min at room temperature, washed twice with PBS and stained with Harris Hematoxylin Solution (Sigma-Aldrich) for 45 min at room temperature. Inserts were washed several times with tap water until the membrane was clean. The membrane was peeled off the plastic inserts, placed on glass microscope slides and mounted using HistoChoice mounting media (Amresco, Solon, OH).

Migrating cells were examined by microscopy at ×200 magnification with Olympus IX70 microscope. Pictures were taken of 5 randomly chosen different fields and migrating cells were counted manually. The average of number of migratory cells for each condition was calculated and the differences in the number of cells that migrated through the membrane were calculated.

### Western blot analysis

Cells were grown until approximately 90% confluent and washed twice in cold PBS. Cells were lysed on ice, using RIPA buffer (1% NP40, 0.5% Na deoxycholate, 0.1% SDS, 150 mM NaCl) containing Halt Protease Inhibitor Single-Use Cocktail (100X) (Pierce, Rockford, IL). The protein concentration in these whole cell lysates was measured using the bicinchoninic acid (BCA) Protein Assay (Pierce) and calculated based on the BSA standard curve. Protein (50–80 μg) was prepared for SDS-PAGE by denaturing the lysate in 6X loading buffer containing β-mercaptoethanol and DTT followed by boiling for 5 min. Protein preparations were loaded into 10% SDS-PAGE gels and run for 90 min at 150 V. Proteins on the gel were transferred onto nitrocellulose membrane (Bio-Rad, Hercules, CA) for 1 h at 110 V, followed by washing 3 times in TBS with 0.1% Tween-20. Membranes were blocked in TTBS with 5% non-fat dry milk for 45 min and then incubated overnight at 4°C with polyoclonal antibodies in primary antibody diluent solution from the SuperSignal Western Blot Enhancer kit (Thermo Scientific) containing either anti-Pak2 or anti-β-actin (Cell Signaling Technologies, Danvers, MA). Membranes were washed 3 times in TTBS and incubated for 1 h at room temperature with the appropriate HRP-conjugated secondary antibody (anti-mouse for β-actin, Santa Cruz Biotechnology, Inc.; anti-rabbit for Pak2, Santa Cruz Biotechnology, Inc.). Membranes were washed three times in TTBS and developed by ECL western blotting detection system from Santa Cruz Biotechnology, Inc. Finally, chemiluminescence was detected via exposure to HyBlot CL autoradiography film (Denville Scientific, Metuchen, NJ). Digital images of the membranes were scanned and bands intensities were quantified using ImageJ analysis.

### Small interfering RNA transfections

For the wound healing assays, OV2008 and C13 cells were grown in modified 35-mm tissue culture dishes until 50–60% confluent. Cells were transfected with 10 μl of TransIT-TKO transfection reagent (Mirus Bio LLC, Madison, WI) and either 50 nM non-targeting control siRNA or 50 nM SignalSilence Pak2 siRNA (Cell Signaling Technology). The siRNA sequences are proprietary. The transfection was performed in antibiotic-free media and the cells were incubated for 24 h before changing the media. When the cells reached confluence (usually one additional day) the wound was created with a 10-μl pipette and time point zero was photographed with the IX70 microscope. Cells were then incubated for 20 h at 37°C and 5% CO_2_ until time point 20 h was then captured. Mock transfection with TransIT-TKO reagent alone revealed that the transfection process decreased cell movement in general. Therefore longer time periods were used to assess wound healing and migration assays post-transfection.

For the migration assays, cells were first plated in 35-mm dishes and allowed to attach overnight. The next day, cells were transfected with either control siRNA or Pak2 siRNA. The media was changed after 24 h of incubation. Forty-eight hours post-transfection, the cells were trypsinized, counted and seeded into the inserts as described above. Cells were allowed to migrate for 48 h before the inserts were stained, washed and mounted on slides as described above.

### Microarray

Cells were plated and cultured on uncoated, fibronectin-coated or collagen I-coated glass cover slips for 24 h. Total RNA of the cells was then isolated using the RNeasy Mini kit (Qiagen). RNA yield was determined using the ND-1000 spectrophotometer (NanoDrop). The Ambion WT Expression kit (Ambion) was used to synthesize first-strand cDNA from the isolated RNA. Next, a template for transcription was created by synthesizing second-strand cDNA. Antisense cRNA was synthesized and amplified by *in vitro* transcription in a thermal cycler (Eppendorf Mastercycler Gradient). The resulting cRNA was purified using nucleic acid binding beads. Next, second cycle cDNA was synthesized by the reverse transcription of cRNA using random primers before hydrolyzing the cRNA template with RNase H. Second-cycle cDNA was purified using nucleic acid binding beads before the yield was determined using the ND-1000.

The second-cycle cDNA was fragmented and labeled using the GeneChip WT Terminal Labeling kit (Affymetrix). The fragmented and labeled DNA samples were hybridized to a GeneChip Human Gene 1.0 ST Array cartridge for 17 h at 45°C at 60 rpm in a GeneChip Hybridization Oven 640 (Affymetrix). The chips were then stained and washed using the GeneChip Hybridization, Wash and Stain kit (Affymetrix) and a GeneChip Fluidics Station 450 (Affymetrix). Finally, the chips were scanned using the Affymetrix GeneChip Scanner 3000 using Affymetrix GeneChip Command Console software. The resulting cell files were analyzed using BRB-ArrayTools developed by Dr Richard Simon and BRB-ArrayTools Development Team. JMP Genomics Suite 6.0 was also used to analyze and graph the results.

### Statistical analysis

Means derived from multiple treatments in the two ovarian cancer cell lines were analyzed by one way analysis of variance (ANOVA) using SPSS, version 20. Tukey and LSD were the two post-hoc tests used. In all cases, p<0.05 was considered significant.

## Results

### Migration of OV2008 and C13 cells

**Figure 2:**
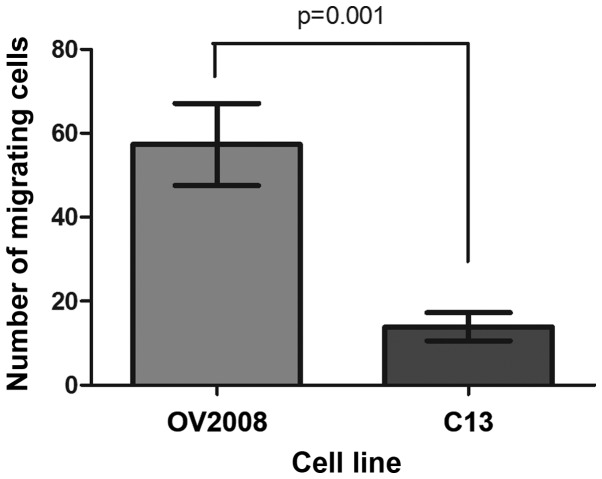
OV2008 cells migrate better through an uncoated 8 μm porous membrane than their cisplatin-resistant sister cell line, C13. Values are presented as % of open area and represent the mean ± SEM; OV2008 n=9, C13 n=5.

Migration through the porous membrane of transwell inserts was used to measure 3-D motility of the selected cell lines. More than 3 times as many OV2008 cells migrated through the cell culture insert membranes than C13 cells over a 24-h period and the difference was significant (Fig. 2).

**Figure 3:**
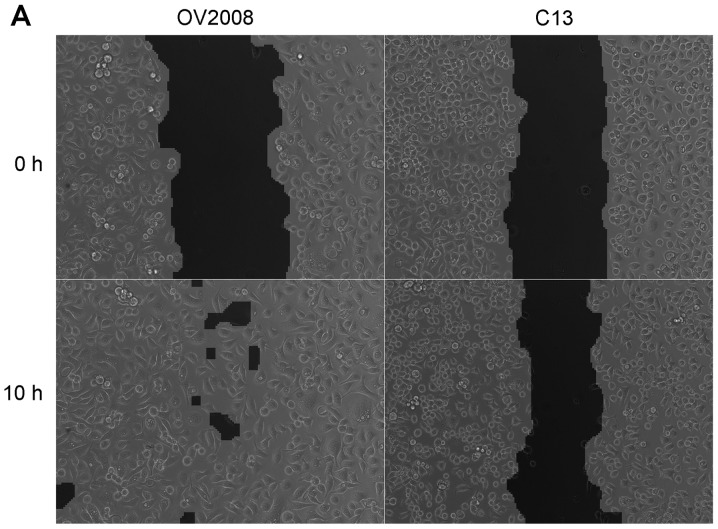

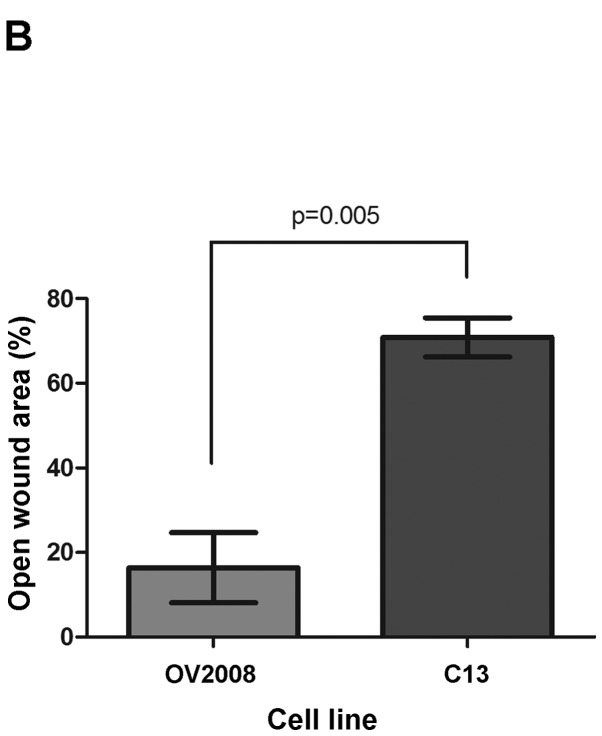
OV2008 cells heal a scratch wound better than the C13 cells on uncoated glass cover slips. (A) Scratch wound assay for OV2008 and C13 ovarian cancer cell lines. Cells were plated on glass cover slips for 24 h prior to scratch wounding to remove cells. The cells were cultured for an additional 10 h and monitored with time-lapse microscopy. The images are representative samples of images obtained at 0 and 10 h after the scratch. (B) Migratory capacity of OV2008 and C13 cells. Wound healing assay was performed 24 h after cells were seeded on glass cover slips. Migration potential was measured by scratching the confluent monolayer and observing the movement of cells into the gap. Values are presented as % of open area and represent the mean ± SEM; OV2008 n=4, C13 n=3.

Removing cells from a confluent culture creates a scratch wound (Fig. 3A). Although the overall area of the wound is slightly greater for OV2008 cells, the wound is almost completely closed 10 h post-wounding, whereas the wound for the C13 cells remained substantially open during the same time period. Thus, the OV2008 cells had a much greater capacity to heal a scratch wound than the C13 cells when cultured on untreated glass cover slips (Fig. 3B). When the amount of wound remaining open 10 h post-wounding was compared as a percentage of the area of the original wound (0 h), there was over 3-fold more open wound area remaining in C13 than OV2008 cells (Fig. 3A).

**Figure 4:**
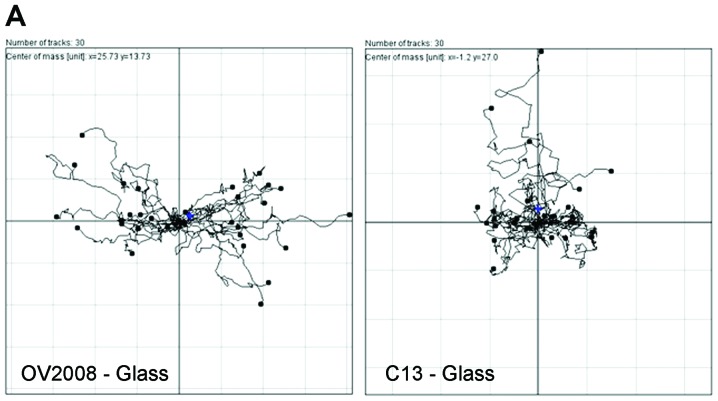

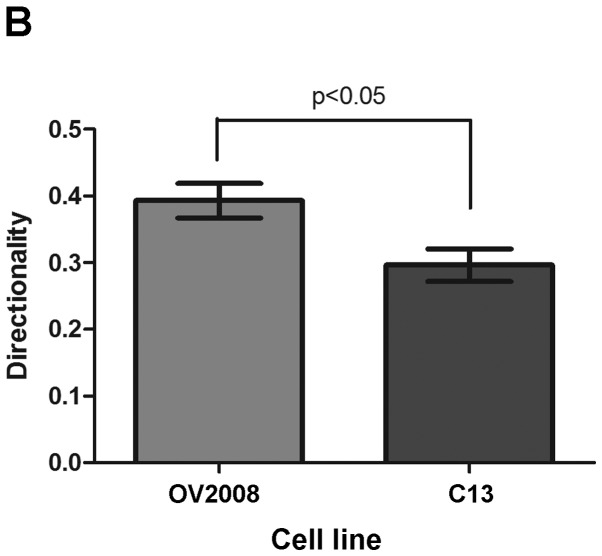
Directionality of OV2008 and C13 cells cultured on glass. (A) Cells were tracked every 10 min from 0–10 h post-wounding and their paths overlayed on a polar grid. Paths are oriented such that the start point is normalized to the origin and the wound face runs parallel to the y-axis. Each plot represents 10 individual cell tracks from 3 separate experiments (30 total cell tracks). (B) Directionality Index (Euclidean Distance/Accumulated Distance) of OV2008 and C13 cells. Values represent the mean ± SEM, n=30.

In order to determine if the wound was being closed due to cell movement into the wound, individual cells randomly selected along the border of the wounds were tracked in each frame of the time-lapse recordings. Inspection of the time lapse recording reveals that the OV2008 cells do migrate into the open area to heal the wound whereas the C13 cells do not generally move into the wound. Somewhat surprisingly, both cell lines have highly motile cells. The OV2008 cells primarily moved perpendicular to the edges of the wound into the open area to effectively close the wound. In contrast to the OV2008 cells, the C13 cells did not move towards the open area, rather C13 cells tended to move parallel to the wound edges and to often change direction. Although the C13 cells were motile, the cell movement did not result in wound closure. When the trajectory of cells was plotted on a polar coordinate grid (Fig. 4), a directionality index (Euclidean distance/total distance moved) can be developed where an index of 1.0 represents completely directional movement in a straight line perpendicular to the wound edge and an index of 0.0 represents totally random cell movement. There is a 1.3-fold greater tendency of the OV2008 cells to move in a directional fashion than for C13 cells. Thus, as measured by two different motility assays, OV2008 cells appear to have a greater capacity for directional movement as compared to the C13 cell line.

### Collagen type I enhances the migration of ovarian cancer cells

**Figure 5:**
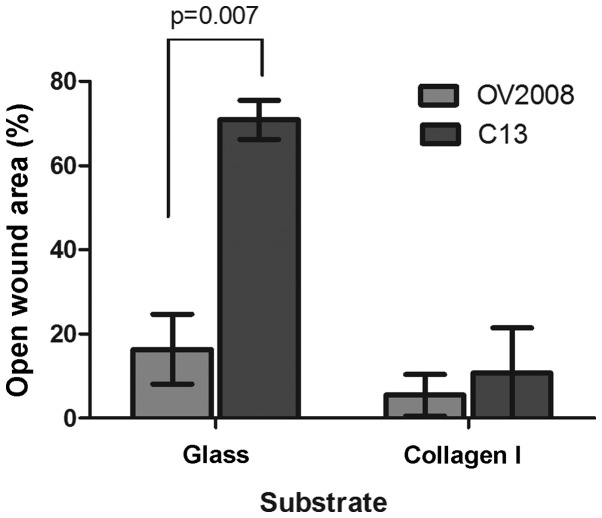
Culturing cells on collagen I increases migration in OV2008 and C13 cell lines. Wound healing assay was performed 24 h after cells were seeded on uncoated or collagen I-coated cover slips. Migration potential was measured by scratching the confluent monolayer and observing the movement of cells into the gap. Cells cultured on glass showed a greater open wound area compared with cells grown on collagen I at 10 h after the wound was made. Values are presented as % of open area and represent the mean ± SEM; OV2008 n=4, C13 n=3.

The effect of collagen I on the migratory behavior of the OV2008 and C13 cells plated on collagen I-coated cover slips is illustrated in Fig. 5. Although there was a slight decrease in the amount of wound remaining open 10 h post-wounding when compared as a percentage of the area of the original wound (0 h) for cells plated on collagen I compared to cells plated on glass for the OV2008 cells, the difference was not significant. In contrast, collagen I significantly increased total wound healing motility in C13 cells such that only about 10% of the wound area remained open. Furthermore, there was no longer a wound healing difference between the two cell lines and thus collagen I makes the two cell lines phenotypically similar in regard to migratory capacity.

**Figure 6:**
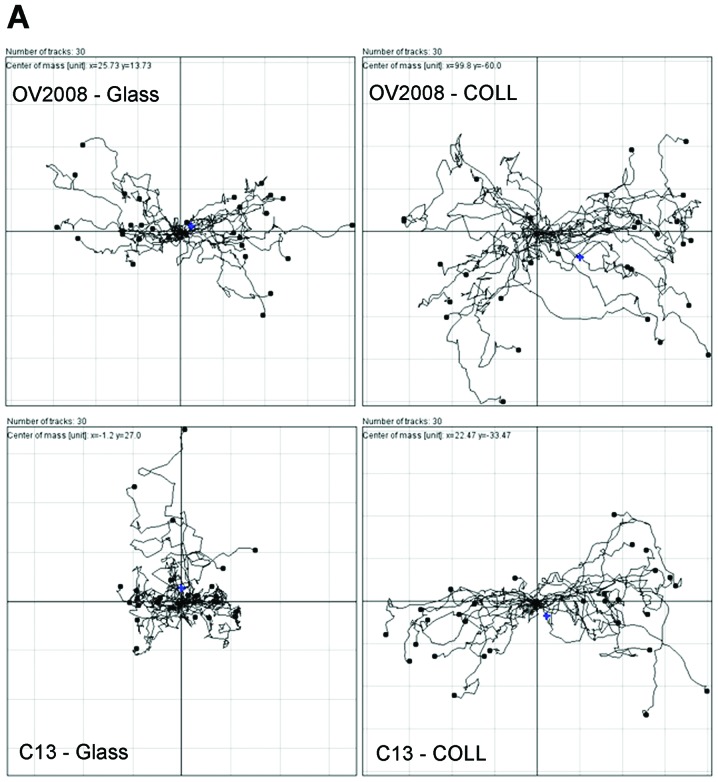

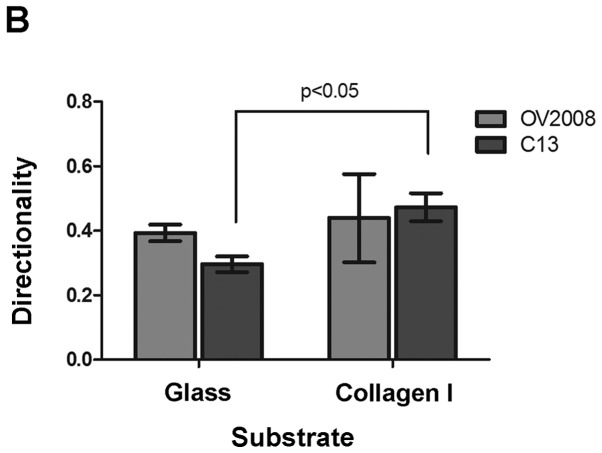
Directionality of OV2008 and C13 cells cultured on glass and collagen I. (A) Cells were tracked every 10 min from 0–10 h post-wounding and their paths overlayed on a polar grid. Paths are oriented such that the start point is normalized to the origin and the wound face runs parallel to the y-axis. Each plot represents 10 individual cell tracks from 3 separate experiments (30 total cell tracks). (B) Directionality Index (Euclidean Distance/Accumulated Distance) of OV2008 and C13 cells on glass and collagen I. Values represent the mean ± SEM, n=30.

Analysis of time lapse recordings for directional cell movement demonstrates that as with wound healing, collagen I did not alter the directionality of OV2008 cells during wound healing (Fig. 6). In contrast, the directionality of individual cells during wound healing was increased in C13 cells by collagen I as compared to glass (Fig. 6A) as was the directionality index (Fig. 6B). Thus collagen I allows C13 cells to express a migratory behavior similar to that of OV2008 cells.

### Fibronectin reduces the migratory capacity of OV2008 cells

**Figure 7:**
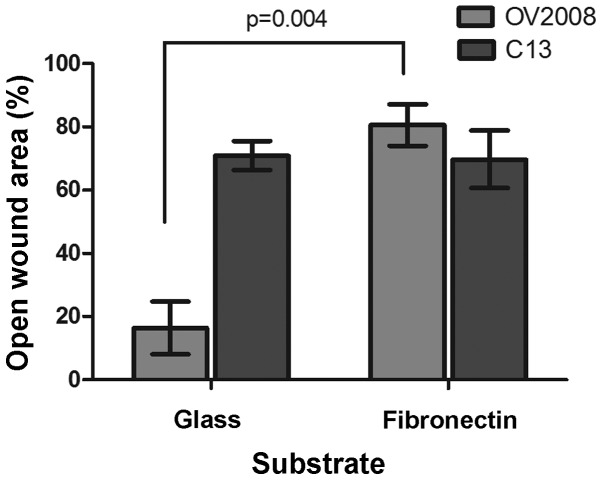
Culturing cells on fibronectin decreases migration in OV2008 but not C13 cells. Wound healing assay was performed 24 h after cells were seeded on uncoated or collagen I-coated cover slips. Migration potential was measured by scratching the confluent monolayer and observing the movement of cells into the gap. OV2008 cells cultured on fibronectin showed greater open wound area compared with cells cultured on glass at 10 h after the wound was made. Wound healing of C13 cells was not affected by the presence of exogenous fibronectin. Values are presented as % of open wound area and represent the mean ± SEM; OV2008 n=4, C13 n=3.

OV2008 and C13 cells cultured on fibronectin-coated cover slips move differently than on those coated with collagen I. In contrast to collagen I, fibronectin significantly reduced OV2008 total wound healing motility as illustrated in Fig. 7 and there was essentially no effect of fibronectin on C13 total wound healing motility. So, OV2008 cells cultured on fibronectin- coated glass appeared to express a phenotype similar to C13 cells migrating on uncoated glass or fibronectin.

**Figure 8:**
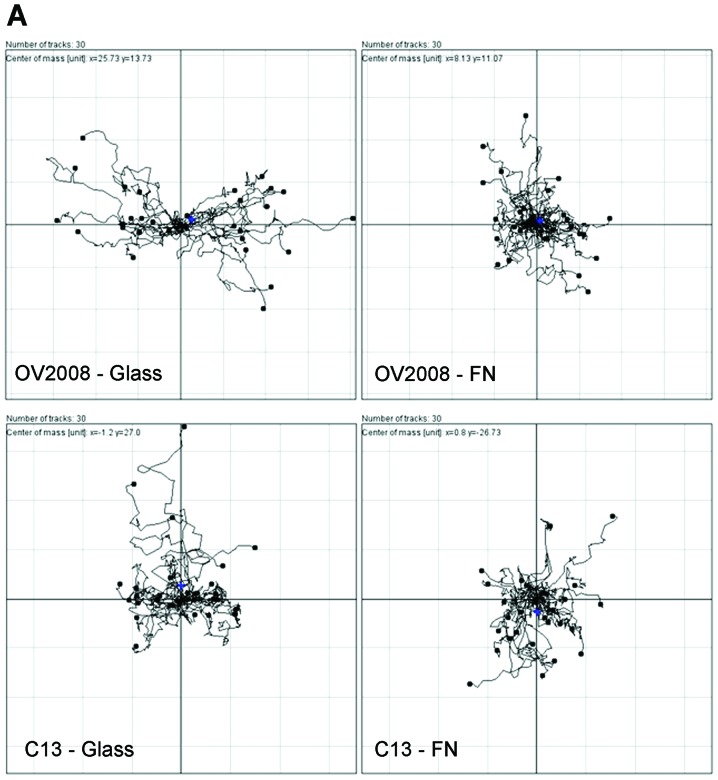

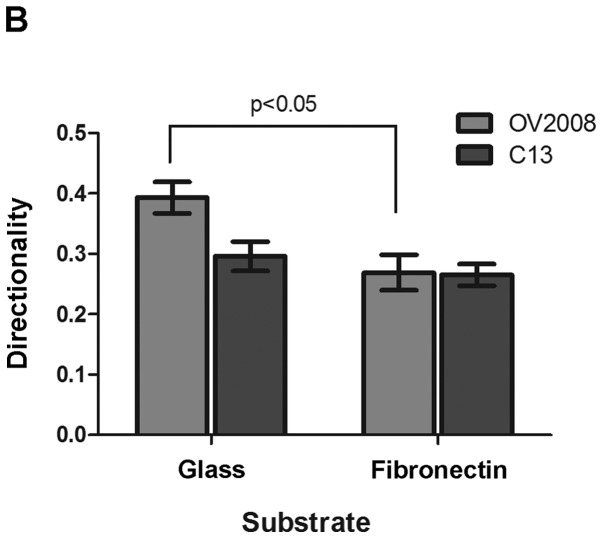
Directionality of OV2008 and C13 cells cultured on glass and fibronectin. (A) Cells were tracked every 10 min from 0–10 h post-wounding and their paths overlayed on a polar grid. Paths are oriented such that the start point is normalized to the origin and the wound face runs parallel to the y-axis. Each plot represents 10 individual cell tracks from 3 separate experiments (30 total cell tracks). Cells tracks were recorded using ImageJ and the following plug-ins: Manual Tracking, Ibidis Chemotaxis and Migration Tool. (B) Directionality Index (Euclidean Distance/Accumulated Distance) of OV2008 and C13 cells on glass and fibronectin. Values represent the mean ± SEM, n=30.

In terms of directionality as determined from time lapse microscopy, the effect of fibronectin was opposite of that for collagen I, such that the directionality of C13 cells was not changed when the cells were cultured on fibronectin and the directionality of individual cells during wound healing was decreased for OV2008 cells by fibronectin as compared to glass (Fig. 8).

**Figure 9:**
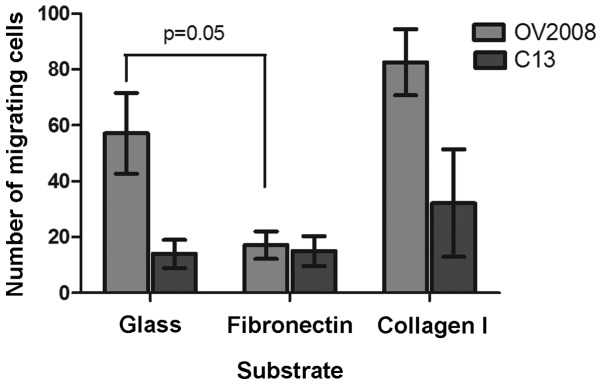
Culturing cells on collagen I and fibronectin affects migration in OV2008 and C13 cells. Cells (2×10^5^) were placed in the upper chamber of uncoated, collagen I-, or fibronectin-coated membranes and allowed to migrate for 24 h. Cells that migrated from the upper to the lower chamber were stained with hematoxylin and pictures taken with Olympus IX70 microscope. Cells from 5 different fields/images were counted from 3 different experiments and data are presented as average number of migratory cells. Values represent the mean ± SEM, n=5.

In addition to the effect of collagen I and fibronectin on wound healing and directionality, migration across a membrane was effected. There was a significant 65% reduction in the number of OV2008 cells that migrated through the fibronectin-coated membrane than through the uncoated membranes as illustrated in Fig. 9. There was no effect of the fibronectin coating on migration of C13 cells through the insert membranes. In contrast and consistent with the effect observed in the wound healing assays, collagen type I increased the migration of the cells through the culture insert membranes such that there was a 2.3-fold increase in the number of C13 cells that migrated through the collagen I coated membranes than through the uncoated membranes as illustrated in Fig. 9. Although not statistically significant, there also was a 1.7-fold increase in the number of OV2008 cells migrating through the collagen I coated membranes. Thus, two different motility assays showed that fibronectin decreases and collagen I increases the migratory ability of the OV2008 and C13 cells.

### Identification of relevant genes

**Figure 10:**
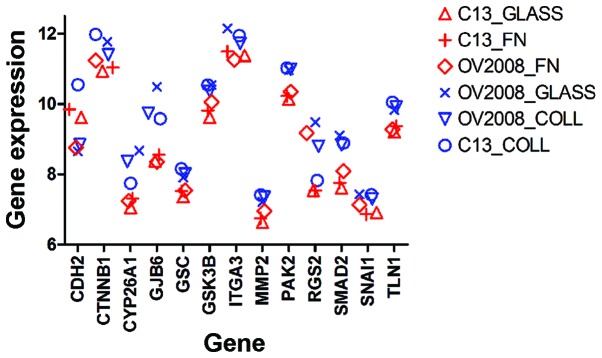
Overlay plot of gene expression grouped by phenotype (more motile cells are blue = OV2008-Glass, C13-Coll-I, OV2008-Coll I; less motile cells are red = C13-Glass, OV2008-FN, C13-FN).

Exploratory analyses of gene expression in cells expressing the different cell movement phenotypes observed when the cells were cultured on glass, collagen I or fibronectin, were performed for each of the cell lines for each of the above conditions. Treatments were divided by phenotype into two groups: motile/directional cells (OV2008-Glass, OV2008-Collagen, C13-Collagen) and the less motile/less directional cells (C13-Glass, C13-Fibronectin, OV2008-Fibronectin) and it was apparent that there was differential expression of several genes known to play a role in cell motility. Treatments were divided by phenotype into two groups (Fig. 10): motile cells (OV2008-Glass, OV2008- Collagen, C13-Collagen) and the less motile cells (C13-Glass, C13-Fibronectin, OV2008-Fibronectin).

### Pak2 inhibition and knockdown reduces the ability of ovarian cancer cells to migrate

**Figure 11:**
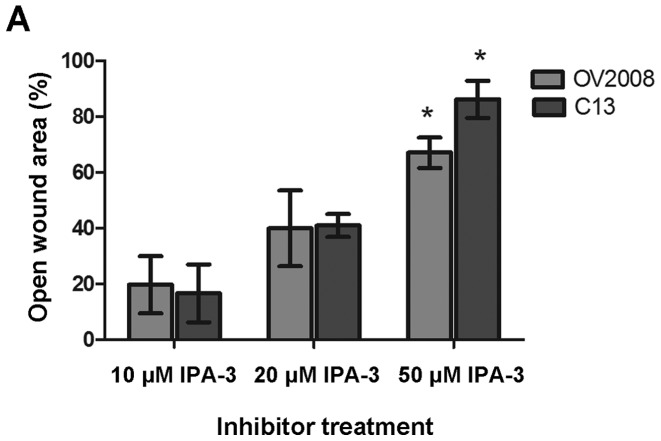

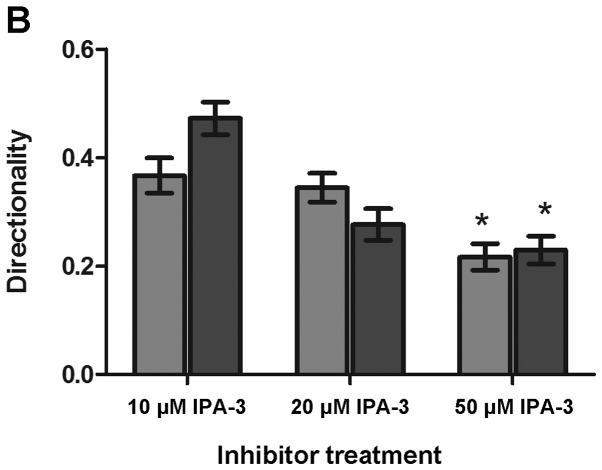
Effect of IPA-3 on wound healing and directionality of OV2008 and C13 cells. Group I Pak inhibition decreases OV2008 and C13 migration potential on collagen I. (A) Wound healing assay was performed 2 h after treatment with IPA-3 at the indicated concentrations. Migration potential was measured by scratching the confluent monolayer and observing the movement of cells into the gap. Cells treated with IPA-3 showed more open wound area compared with untreated cells at 10 h after the wound was made. Values are presented as % of open area. (B) Confluent monolayers of cells were treated with the indicated concentration of IPA-3 2 h prior to wounding. Cells were tracked every 10 min from 0–10 h post-wounding and directionality was calculated by comparing the Euclidean and accumulated distances of the cells. Values represent the mean ± SEM, n=30.

Among the candidate shown in Fig. 10, p21-activated kinase 2 (PAK2) provided tight grouping and distinct separation of the motility phenotypes and has been reported to be either upregulated or hyperactivated in a variety of human cancers. In order to determine the role of PAK in the migratory changes that were observed when the cells were grown on collagen type I, both the OV2008 and C13 cells were pretreated with the PAK inhibitor, IPA-3, that targets the auto-regulatory mechanism of group I PAKs. When C13 cells were cultured on collagen I and treated with increasing concentrations of the PAK inhibitor (IPA-3) for 2 h prior to the wound healing assay, there was a dose-dependent response such that there was increase in the percent of open wound area with an increasing inhibitor concentration (Fig. 11A). There was a similar effect on wound healing in OV2008 cells cultured on collagen I and treated with increasing concentrations of IPA-3.

As shown in Fig. 11B, when C13 cells were cultured on collagen I and treated with increasing concentrations of IPA-3 for 2 h prior to the wound healing assay, there was also a dose-dependent response in terms of individual cell directionality. Directionality of OV2008 cells was similarly affected. Thus, inhibition of PAK prevented the motility/directionality promoting effect of collagen I on cells.

**Figure 12:**
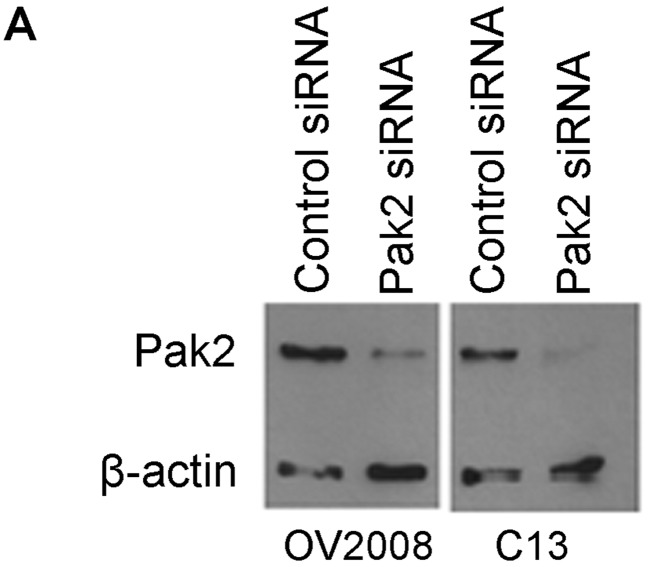

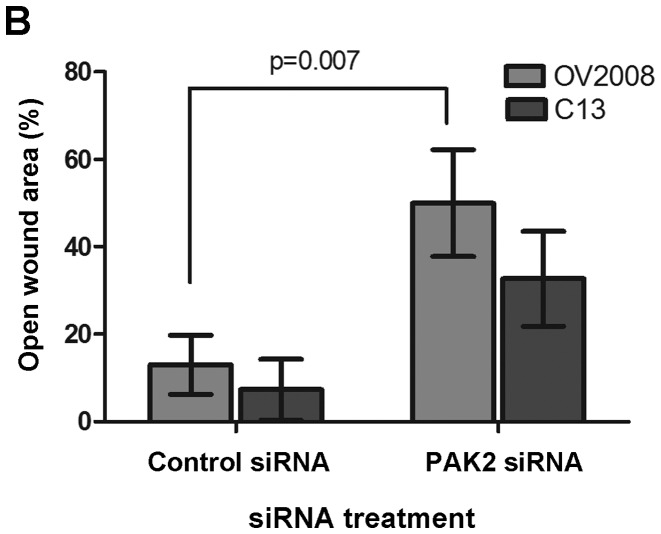

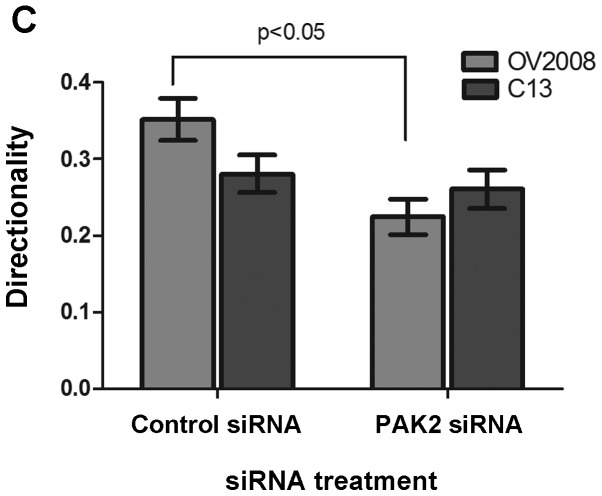
Specific knockdown of Pak2 using siRNA transfection reduces both wound healing ability and directionality of migrating ovarian cancer cells. (A) Western blotting was used to verify that Pak2 protein was effectively knocked down following transfection with Pak2 siRNA. (B) Wound healing assay was performed 24 h after cells were seeded on collagen I-coated cover slips. Cells transfected with Pak2 siRNA showed a greater open wound area compared with cells transfected with control siRNA at 20 h after the wound was made. Values are presented as % of open area, n=3 (C) Directionality Index (Euclidean Distance/Accumulated Distance) of OV2008 and C13 cells post-transfection. Values represent the mean ± SEM, n=30.

Since there appeared to be an effect of IPA-3 inhibition on both OV2008 and C13 cell migration, siRNA transfection was used to specifically inhibit the Pak2 isoform of PAK prior to wound healing assays on collagen I-coated coverslips. Western blotting was used to verify that Pak2 protein was effectively knocked down following transfection with Pak2 siRNA (Fig. 12A). When the mean amount of wound remaining open 20 h post-wounding was compared as a percentage of the area of the original wound (0 h), there was a significant 4-fold greater open wound area remaining in OV2008 cells receiving Pak2 siRNA than the cells receiving control siRNA (Fig. 12B). Although there was a similar 4-fold greater open wound area remaining in C13 cells receiving Pak2 siRNA than the cells receiving control siRNA, the difference did not achieve statistical significance (Fig. 12B). Additionally, transfection with Pak2 siRNA significantly decreased the ability of OV2008 cells to move in a directional manner on collagen I (Fig. 12C). However, the directionality of C13 cells on collagen I was not affected by Pak2 knockdown.

## Discussion

The current studies investigated the motility characteristics of two related ovarian cancer cell lines (OV2008 and C13) and found that OV2008 cells had a much greater capacity to migrate through a porous membrane of a transwell insert and heal a scratch wound when cultured on glass than the C13 cells. Furthermore, the studies indicate that wound healing is due to characteristics associated with cell movement rather than cell proliferation and specifically attributable to a difference in directionality as opposed to differences in the total distance that the cells moved. These differences were unexpected because the C13 cells were selected to have reduced chemo-sensitivity via a series of *in vitro* cisplatin challenges of the parental OV2008 cell (23) with no attention given to changes in motility. In subsequent experiments, these differences in cell motility could be negated or exaggerated by culturing the cells on one ECM substrate versus another.

Culturing cells on collagen I coated cover slips or transwell membranes increased total wound healing and individual cell directionality, as well as migration through a porous membrane for both cell lines with the effect being the most dramatic on the migration of C13 cells. While the two cell lines migrated differentially on uncoated glass, they become phenotypically similar when plated on collagen I coatings. These findings are consistent with previous reports in the literature which used collagen I as a chemo- and haptotactic attractant for ovarian cancer cells. Recently, Shen *et al* (24) observed that ovarian cancer cells demonstrate enhanced migration through a collagen type I-coated transwell invasion assay. Similarly, Ahmed *et al* (25) showed that collagen I enhanced the migration of ovarian cancer cell lines through a Boyden chamber when collagen I was used as a chemo-attractant in the lower part of the chamber as well as when it coated the upper part of the chamber. The current studies confirm these findings and further reveal that the effect of collagen type I on the migration of ovarian cancer cells is at least in part due to an increase in directionality. Although the complex process of cellular motility is well studied and it is known that cells must polarize, protrude, adhere and reorganize the cytoskeleton in order to move, it is not clearly understood how the basic motility machinery is coupled to a steering mechanism that integrates environmental cues such as ECM (26). By showing in the present investigation that collagen I provides ‘steering’ cues to migrating ovarian cancer cells, we can better understand how collagen type I facilitates cancer cell movement.

Understanding the effect of collagen type I is important because ovarian tumor cells have been shown to attach preferentially at locations where the mesothelium is disrupted and the underlying collagen I-rich stromal matrix is exposed (27). Collagen type I is abundant in the interstitial matrix and therefore is a substrate that ovarian cancer cells disseminated to the peritoneal cavity will inevitably encounter upon attachment and exposure to the submesothelial ECM during invasion (28). Moser *et al* (29) demonstrated that ovarian carcinoma cells adhere preferentially to type I collagen and Burleson *et al* (28) reported that ovarian cancer spheroids can completely disaggregate on type I collagen coatings. Both of these studies examined disaggregation of cells from spheroids on collagen type I, so the effect of collagen I on cell motility was only indirectly inferred, whereas the current studies tracked individual cells and provide direct measures of distance and direction for cell movement in response to being cultured on collagen I.

In contrast to the effects of collagen I on cell movement, in the current investigation fibronectin (FN) reduced the migration of OV2008 cells as measured by two different migration assays. While the two cell lines migrated differentially on uncoated glass, they become phenotypically similar when plated on fibronectin coatings, adopting the movement characteristic of C13 on glass. That is, the cells migrate less directionally on FN than on collagen type I or uncoated glass. Fibronectin is another important constituent of the ECM at the metastatic site of ovarian cancer. Mesothelial cells express fibronectin on the apical surface and soluble FN has been detected in the ascities fluid from ovarian cancer patients. However, the impact of fibronectin on ovarian cancer cells is less clear than that of collagen I with evidence for both increased and decreased movement in association with FN. It has been reported that FN released by peritoneal mesothelial cells stimulates ovarian cancer cell motility (30). In a study by Ahmed *et al* (25), fibronectin increased the migration of ovarian cancer cells through a Boyden chamber when used as a chemoattractant in the lower part of the chamber. Fibronectin has been reported to increase the migration of pancreatic (31) and breast cancer (32) cells as well. On the other hand, there is evidence indicating that metastatic potential is inversely correlated with FN expression (33,34). Also, a correlation between the loss of FN and acquisition of the malignant phenotype *in vitro* and of tumorigenic and metastatic phenotypes *in vivo* has been reported (35,36).

It is possible that migration on fibronectin is mediated by other proteins that are differentially expressed in OV2008 and C13 cells. Jiao *et al* (37) recently showed that human melanoma cell migration on fibronectin was greatly decreased when matrix metallopeptidase-2 (MM P-2) activity was inhibited. The authors (37) suggested that active MMP-2 is recruited to the leading edge of invasive tumor cells and cleaves fibronectin into shorter fibronectin products. The fibronectin fragments promote αvβ3 integrin recruitment to the area of cleaved fibronectin products to facilitate tumor cell adhesion and migration. Interestingly, MMP-2 was identified in the current investigation via microarray as being expressed higher in the more motile cells than in the less motile cells and the expression was decreased by FN in both cell lines. An elegant study by Kenny and Lengyel (38) used several different models to examine the expression of MM P-2 in ovarian cancer cells. In one model, a novel organotypic 3D coculture model mimicking human omentum was created using collagen I, human primary mesothelial cells (HPMCs), and human primary fibroblasts (HPFs) that were extracted from human omentum. The relative expression of MM P-2 mRNA was 20-fold higher in ovarian cancer cells attached to the 3D coculture than in ovarian cancer cells adhering to plastic. Thus it is possible that in the current study that the differential expression of MMP-2 influenced the ability of cells to interact with the FN and their ability to migrate on fibronectin-coated surfaces.

The results of the migration assays in the present investigation clearly demonstrated that collagen I and fibronectin modify the migratory process of ovarian cancer cells and microarrays were used to study gene expression patterns and narrow down the list of possible mediators. When comparing the gene expression profiles of OV2008 and C13 cells grown on glass, collagen I and fibronectin, a relatively short list of candidate genes was established. Among the candidates, p21-activated kinase has been reported to be either upregulated or hyperactivated in a variety of human cancers such as breast, ovary, colorectal, thyroid and pancreatic (21). In addition, it is activated by the Rho family GTPases Cdc42, Rac and Rho, which are activated in response to ECM adhesion (39).

Initial screening for the role of PAKs in mediating directional movement of OV2008 and C13 cells was accomplished using the group I PAK (PAK1, PAK2, PAK3) chemical inhibitor, IPA-3 which was first identified as a highly selective inhibitor of group I PAK kinases by testing its effect on over 200 different kinases (40). It was reported that this selectivity was achieved by targeting the distinct autoregulatory mechanism conserved in group I PAKs (40). In the present study, inhibition of group I PAKs with IPA-3 resulted in decreased total wound healing and directionality of OV2008 and C13 cells grown on collagen I and confirmed a role for one or more of the group I PAKs. While a majority of the literature is focused on the Pak1 isoform, both Pak1 and Pak2 have been implicated in cell motility. Coniglio *et al* (41) reported that Pak2 was needed to generate new focal adhesions and to limit the sizes of focal adhesions in breast cancer cells. In prostate cancer cells, Bright *et al* (42) observed that both Pak1 and Pak2 affected migration speed. Knockdown of Pak1 and Pak2 in ovarian cancer cell lines reduced cell migration and invasion (43). Pak2 has also been implicated in the migration of hepatocellular carcinoma cells (44) and monocytes (45). In order to specifically assess the role of the Pak2 isoform, which was identified as a candidate in the microarray experiments, knockdown of Pak2 with an isoform specific siRNA sequence was performed and resulted in decreased overall cell migration as well as decreased directionality of individually migrating cells.

Pak2 appears to play a role in generating new focal adhesions as well as limiting the sizes of focal adhesions. Pak2-depleted breast cancer cells contain significantly larger focal adhesions and are unable to generate new focal adhesions (41). During cell migration, the assembly, maturation, translocation and disassembly of focal adhesions mediate, respectively, cell attachment, contraction, protrusion of leading edges and retraction of trailing edges (46). Therefore, by controlling the generation and size of focal adhesions, Pak2 may play an important role in the regulation of cell migration. Thus the loss of general movement and directionality in cells that were transfected with Pak2 siRNA could be due to unrestricted, large focal adhesions anchoring the cells to the substrate which would prevent any movement of the cells. Another of our candidate genes, talin-1 (TLN1), would be worthy of investigation in the future. TLN1 has been shown to play important roles in adhesion, cytoskeletal organization and regulation of integrin signals during cell migration (47).

In the traditional analysis of wound healing assays, the width of a scratch is compared at time point 0 to that at the end of the experiment. When the wound has not healed, the cells are assumed to not have migrated (48). However, time lapse microscopy revealed that even when the cells were not healing the wound, they were indeed moving. By tracking individually migrating cells, we were able to measure the distance and direction of the cells moved and show that collagen I has the ability to direct cell movement into the wound and thus promote healing. In contrast, fibronectin was able to decrease directionality in OV2008. Directed cellular migration is important in facilitating the coordinated movement of cells throughout development and in wound repair (49). Directional movement may also affect the ability of metastasizing ovarian cancer cells to colonize the peritoneal microenvironment. The ability of fibronectin to inhibit metastasis in some studies may be in part to the effect on directionality. Finally, our results also suggest that the use of PAK inhibitors should be explored for possible use in conjunction with tumor debulking to limit the spread of ovarian metastases throughout the peritoneal cavity.
